# Effect of Menopausal Hormone Therapy on Cellular Immunity Parameters and Cytokine Profile

**DOI:** 10.3390/biomedicines12081892

**Published:** 2024-08-19

**Authors:** Marina Averyanova, Svetlana Yureneva, Viktoriia Kiseleva, Oksana Yakushevskaya, Marina Iskusnykh, Anna Pavlova, Andrey Elchaninov, Timur Fatkhudinov, Natalia Mikhanoshina, Tatiana Ivanets, Valentina Vtorushina, Lyubov Krechetova, Polina Vishnyakova, Gennady Sukhikh

**Affiliations:** 1 National Medical Research Center for Obstetrics, Gynecology and Perinatology Named after Academician V. I. Kulakov of Ministry of Healthcare of Russian Federation, 117997 Moscow, Russiavictoria.kurnosova.1991@gmail.com (V.K.); elchandrey@yandex.ru (A.E.);; 2 Research Institute of Molecular and Cellular Medicine, Peoples’ Friendship University of Russia (RUDN University), 117198 Moscow, Russia; 3 Avtsyn Research Institute of Human Morphology of Federal State Budgetary Scientific Institution “Petrovsky National Research Centre of Surgery”, 117418 Moscow, Russia

**Keywords:** menopausal hormone therapy, immune system, immunity, estrogens, menopause

## Abstract

Background: A woman’s entry into the menopause period is associated with a number of changes in the body, including those related to the immune system. Immune aging is a consequence of age-related changes in the function of immune cells and the composition of their subpopulations. Menopausal hormone therapy (MHT) is thought to partially neutralize the negative effects of aging on the immune system. Objective: We aimed to evaluate the effect of oral and transdermal MHT on cellular immunity parameters and cytokine profile in menopausal women. Methods: Fifty peri- and early postmenopausal women were included. Immune parameters were assessed by flow cytometry and multiplex analysis. Results: We showed that different routes of MHT administration led to significant changes in monocyte phenotype and a decrease in monocyte chemoattractant protein-1 (MCP-1) level in menopausal patients. In addition, oral MHT resulted in a significant increase in NK and B cells. A significant increase in the number of T-helper cells was observed with transdermal MHT. In addition, oral MHT resulted in a significant decrease in IL-1β level. Conclusions: We have demonstrated for the first time that oral therapy, in contrast to transdermal therapy, has a more pronounced effect on specific immune subpopulations of blood cells in menopausal women. This effect is likely to be responsible for its anti-aging properties in the context of immune aging as well as its protective effects in infectious diseases. Perhaps testing blood immune parameters or assessing immune status before prescribing MHT could become a routine step in clinical practice before choosing a patient management strategy.

## 1. Introduction

Menopause is a normal part of a woman’s life. It consists of a series of changes in the body, including the immune system. Immune senescence is a series of structural and functional changes in immune organs and cells. It results in a predisposition to autoimmune diseases and the development of constitutive low-grade inflammation [1].

The immune system does not work alone. The cytokines, hormones and neurotransmitters system integrate the immune, nervous and endocrine systems by sharing receptors on the surface of immunocompetent cells [2].

The decline in ovarian function in peri- and postmenopausal women is thought to play a key role in immune aging [3]. Several studies have shown that postmenopausal women are characterized by increased levels of pro-inflammatory cytokines such as tumor necrosis factor-α (TNF-α), IL-1β, IL-6 and IL-8, decreased levels of anti-inflammatory IL-10 and IL-33 [4], as well as a decreased proportion of naive T cells, accumulation of memory T cells and a significant change in the proportion of CD4+ and CD8+ T lymphocytes [5]. These changes suggest a molecular link between reduced sex steroid levels and immunosenescence [6].

Treatment of the menopausal syndrome is based on compensating for the lack of sex hormones, mainly estrogens. This makes it possible to slow down the progression of the deficiency and thus postpone the onset of organic changes in the target tissues and the main systems of the female body. The aim of menopausal hormone therapy (MHT) is to partially compensate for the sex hormones deficiency, thereby delaying natural aging and offsetting its long-term effects. Studies have shown that MHT partially counteracts the negative effects of aging on the immune system. The use of MHT reduced the elevated plasma levels of the pro-inflammatory cytokines TNF-α, IFN-γ and IL-6 in post-menopausal women [7,8]. Another study showed that the use of MHT was associated with a significant decrease in NK cell cytotoxicity and IL-2 and IFN-γ production [9]. MHT has a beneficial effect on the immune system in menopausal women. This again shows that estrogens play a special role in the regulation of immune functions.

Studying the cellular and cytokine changes that occur during menopause will be important for a more complete understanding of the pathophysiology of immune aging. This will allow precise identification of targets for potential therapy for autoimmune [10,11], oncological [12,13,14], cardiovascular [15,16] and infectious disease prevention [17,18,19] in postmenopausal women.

It is possible that the route of administration of estrogen as part of MHT in women is important in terms of its effect on systemic inflammation. In our study, we attempted to evaluate the effect of different MHT regimens and routes of administration (oral and transdermal) on immune parameters in menopausal women. Few such studies have been published to date, so this study is of particular scientific interest and clinical importance.

## 2. Materials and Methods

### 2.1. Ethics

This study was approved by the Ethics Committee of the Kulakov National Medical Research Center for Obstetrics, Gynecology and Perinatology (Protocol No. 11 dated 11 November 2021). This study was registered on the clinical trials website ClinicalTrials.gov, ID NCT05678192, Protocol ID 11-11/11.2021.

### 2.2. Subjects and Study Design

We examined fifty women in the period of peri- and postmenopause (−1–+1c according to STRAW +10), observed in the Kulakov National Medical Research Center for Obstetrics, Gynecology and Perinatology from December 2021 to March 2023. Exclusion criteria were contraindications to MHT: contraindications to MHT (unexplained vaginal bleeding; breast cancer; suspected estrogen-dependent malignancies of the ovaries and uterus; current or past history of acute and chronic liver disease; current or past history of thrombosis and thromboembolism (including deep vein thrombosis; pulmonary embolism, myocardial infarction, ischemic or hemorrhagic cerebrovascular accidents); submucous uterine myoma; endometrial polyp; allergic reactions to MHT components; cutaneous porphyria; progestogen-dependent neoplasms), obesity; immunodeficiencies; systemic connective tissue diseases; cancer; autoimmune diseases; acute respiratory diseases and exacerbation of chronic diseases within the previous 3 months; use of antibacterial, immunomodulatory drugs or MHT within the previous 3 months. Also, the exclusion criterion was patient refusal to participate in the study at any stage.

The severity of the climacteric syndrome (CS) was assessed using the Greene Climacteric Scale (Table S1 in the Supplementary Materials) [20,21]. The questionnaire consists of 21 questions and contains 4 sections reflecting the impact of different symptom groups on women’s quality of life: psycho-emotional (questions 1–11), physical (questions 12–18), vasomotor (questions 19–20) and sexual symptoms (question 21). The severity of the climacteric syndrome was interpreted on the basis of the total score: 1–11—mild; 12–19—moderate; 20 or more—severe. To evaluate the results of the questionnaire, the total score in each symptom category is divided by the number of questions in the section. Risk factors for cardiovascular disease, breast cancer and postmenopausal osteoporosis, as well as the presence of comorbid conditions in women, were taken into account in the selection of the minimum effective dose of medication. Finally, 27 patients received oral MHT, including 1 mg estradiol in combination with dydrogesterone with 10 mg dydrogesterone in a cyclical regimen daily without a break (for perimenopausal women) or 1 mg estradiol in combination with 5 mg dydrogesterone daily orally (for postmenopausal women). In addition, 23 patients received transdermal MHT containing 1.5 mg estradiol—in the form of 0.06% gel—2.5 g in combination with micronized progesterone in a cyclical (200 mg for 12 days monthly—for perimenopausal women) or continuous (100 mg intravaginal daily continuously—for postmenopausal women) regimen. Blood samples were taken before and after 12 weeks of treatment.

### 2.3. Isolation of Peripheral Blood Mononuclear Cells

To isolate peripheral blood mononuclear cells, 2 mL of whole blood-EDTA was taken into a separate tube, and 8 mL of erythrocyte lysis buffer was added for 10 min. The tube was placed at +4 °C. The sample was then centrifuged at 300× *g* for 5 min +4 °C. The supernatant was removed, and 10 mL of phosphate-buffered saline was added to the sediment, it was centrifuged again and the supernatant was removed. The pellet was resuspended in 1000 µL of autoMACS^®^ Rinsing Solution (Miltenyi Biotec, Bergisch Gladbach, Germany) containing 0.1% MACS^®^ BSA Stock Solution (bovine serum albumin, Miltenyi Biotec, Bergisch Gladbach, Germany) and stored at +4 °C until measurement.

### 2.4. Multiparametric Flow Cytometry Assay

The main blood cell subpopulations such as T killer cells, T helper cells, NK cells, B lymphocytes and monocytes were analyzed by flow cytometry.

For this, PBMC single-cell suspensions at a density of 0.1 × 10^5^ in 100 µL autoMACS^®^ Rinsing Solution containing 0.1% MACS^®^ BSA stock solution were incubated with anti-human monoclonal antibodies from Miltenyi Biotec and Beckman Coulter (Indianapolis, IN, USA) according to the fluorophore compositions shown in Table S2 for 10 min at 4 °C in the dark. After washing with autoMACS^®^ Rinsing Solution at 500× *g* for 5 min, cells were resuspended in 1× PBS and analyzed. All samples were analyzed on a FACSCalibur flow cytometer (Becton Dickinson, Franklin Lakes, NJ, USA). At least 10,000 cells were collected for each sample in the lymphocyte and monocyte gates, which were identified on an arbitrary gate created on a bivariate scatter plot of forward vs. side scattered light signals. For each of the gated cell populations, the percentages of cells were analyzed. Control tubes without fluorophores were used for gate identification during analysis. The main steps of gating are shown in Figure S1. Briefly, we created logistic gates for lymphocytes and monocytes. Monocyte subpopulations were determined by CD14 and CD16 expression levels. For lymphocytes, CD3 negative and positive populations were determined. Among CD3 positive cells, T killers and T helpers were identified by the distribution of CD4 and CD8. Populations of B lymphocyte and NK cell populations were determined by the expression levels of CD19 and HLA-DR for the former and CD16 and CD56 for the latter among CD3 negative cells. Flow cytometry data were either analyzed on BD CellQuest™ (Becton Dickinson, Franklin Lakes, NJ, USA) or exported and analyzed in FlowJo™ v10 software (FlowJo LLC, Ashland, OR, USA).

### 2.5. Bio-Plex Multiplex Immunoassay

The Bio-Plex Human Cytokine Screening Panel (Bio-Rad Laboratories, Hercules, CA, USA) was used to detect 27 cytokines in 12.5 μL of plasma. Screening was performed according to the manufacturer’s protocol.

### 2.6. Statistical Analysis

Statistical analysis was carried out using Statistica 13.5.0 and GraphPad Prism 9 software packages. For all quantitative indicators, conformity to the normal distribution was assessed using the Shapiro–Wilk test. The parametric Student’s *t*-test, the non-parametric Mann–Whitney U-test and the χ^2^ method were used to detect differences between two unrelated sets. To compare the parameters in paired samples (before and after MHT), the parametric Student’s *t*-test for related sets, the non-parametric Wilcoxon *t*-test, and McNemar’s test were used. The critical significance level *p* for testing statistical hypotheses was set at 0.05.

## 3. Results

### 3.1. Patients’ Data

The initial clinical and anamnestic characteristics of the patients before the start of oral and transdermal MHT are shown in Table 1.

Initially, the groups of patients who were prescribed different forms of MHT (oral and transdermal) were comparable in terms of clinical and anamnestic characteristics (age, weight, BMI, the proportion of women smoking, duration of menstruation-free period) and severity of menopausal syndrome according to the Greene scale.

**Figure 1:**
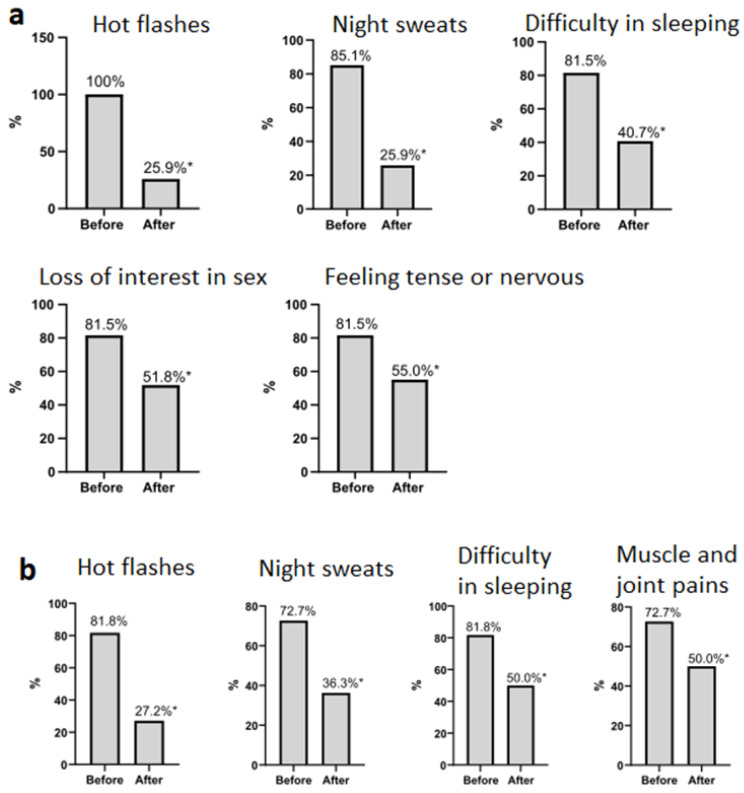
Significant reduction in the incidence of individual menopausal symptoms after 12 weeks of therapy. (**a**) Oral MHT (*n* = 27). (**b**) Transdermal MHT (*n* = 23), * *p* < 0.05 McNemar’s test.

The results of the assessment of the severity and frequency of occurrence of menopausal symptoms at baseline and after 12 weeks of use of oral/transdermal MHT are presented in Table 2, Figure 1a,b.

Table 2 and Figure 1a,b, show that women’s menopausal symptoms, as measured by the Greene Climacteric Scale, decreased significantly in both groups after 12 weeks of oral and transdermal MHT use. This reduction was attributed to a decrease in the severity of psycho-emotional, physical and vasomotor symptoms, as well as sexual dysfunction. Patients in both groups dramatically reduced the frequency of night sweats, hot flashes and sleep disturbances. In addition, the oral MHT group showed a significant reduction in the incidence of symptoms such as tension, anxiety and lack of desire during sex, while the transdermal MHT group showed a significant reduction in the incidence of muscle and joint pain.

### 3.2. Changes in Cellular Immunity Parameters in Women during the Use of MHT

After 12 weeks of oral MHT, patients showed a significant decrease in the number of classical CD14++CD16− monocytes and a significant increase in the number of intermediate monocytes (CD14+CD16++), CD3−CD56+CD16+ NK cells and CD3−CD19+HLA-DR+ B lymphocytes. When analyzing the immune status of patients taking transdermal MHT, a significant increase in the number of T helpers was found. The results are shown in Table 3, Figure S2.

### 3.3. Changes in the Number of Monocyte Subpopulations during the Use of MHT

**Figure 2:**
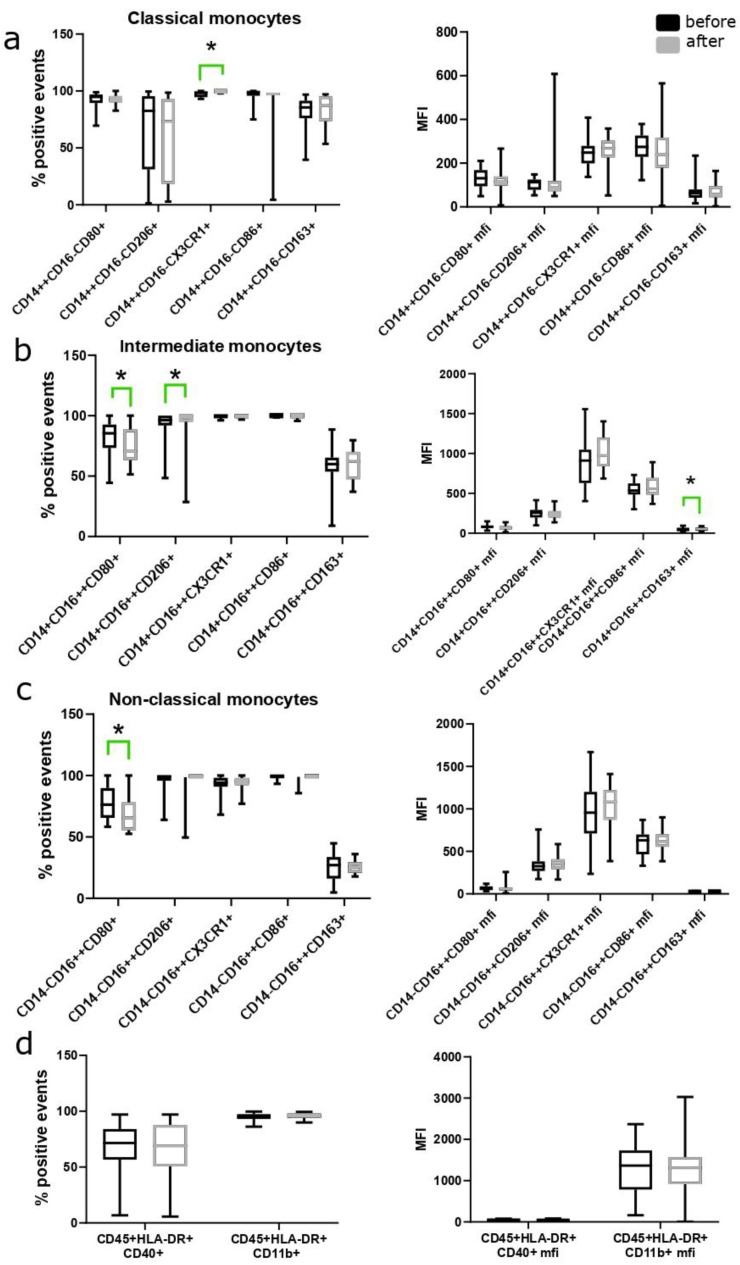
Analysis of the percentage and intensity (MFI) of staining for pro- and anti-inflammatory markers in (**a**) classical (CD14++CD16−), (**b**) intermediate (CD14+CD16++), (**c**) non-classical monocytes (CD14−CD16++) and (**d**) data on monocytes positive for HLA-DR, CD40 and CD11b markers before and after 12 weeks of using transdermal MHT, * *p* < 0.05 according to the Wilcoxon test. Data are presented as median and interquartile range.

**Figure 3:**
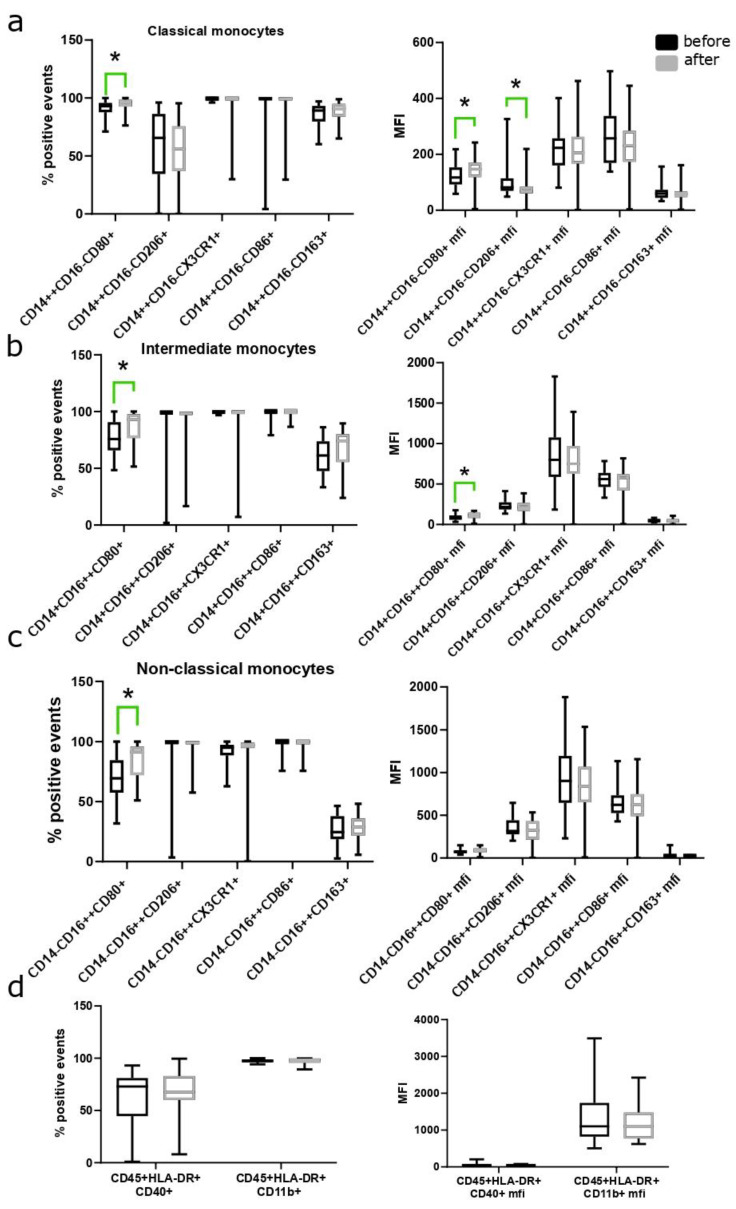
Analysis of the percentage and intensity of staining for pro- and anti-inflammatory markers in (**a**) classical (CD14++CD16−), (**b**) intermediate (CD14+CD16++), (**c**) non-classical monocytes (CD14−CD16++) and (**d**) data for HLA-DR, CD40 and CD11b positive monocytes before and after 12 weeks of oral MHT * *p* < 0.05 Wilcoxon test. Data are presented as median and interquartile range.

Among the monocytic subpopulation, we analyzed the number of cells positive for pro- (CX3CR1, CD86, CD80, CD40) and anti-inflammatory (CD206, CD163) and pan-monocyte markers (CD11b, HLA-DR) and their expression by mean fluorescence intensity (MFI) before and after transdermal (Figure 2) and oral (Figure 3) MHT. No statistically significant differences were found between CD11b and CD40 markers before and after the use of MHT. Among classical (CD14++CD16−) monocytes, transdermal MHT resulted in a statistically significant increase in the level of CD14++CD16−CX3CR1+ cells (*p* = 0.001). The results are shown in Figure 2.

Analysis of the expression levels of pro-inflammatory (CD86, CD80, CD40) and anti-inflammatory markers (CD163, CD206) in classical (CD14++CD16−) and non-classical (CD14−CD16++) and intermediate (CD14+CD16++) monocytes showed a statistically significant increase in the number of CD14+CD16++CD206+ cells (*p* = 0.01), as well as a decrease in the MFI of the CD163 marker in CD14+CD16++ cells (*p* = 0.03), the number of CD14+CD16++CD80+ cells (*p* = 0.0462) and CD14−CD16++CD80+ cells (*p* = 0.031) using transdermal MHT.

Analysis of blood flow cytometry data from patients before and after oral MHT revealed changes in the subpopulation composition of immune cells, namely a significant increase in both the percentage and intensity of staining in classical (CD14++CD16−), intermediate (CD14+CD16++) and non-classical (CD14−CD16++) monocytes positive for the pro-inflammatory marker CD80. A significant decrease in staining intensity was also observed in classical monocytes positive for the anti-inflammatory marker CD206 (Figure 3).

### 3.4. Changes in the Cytokine Profile in Women during the Use of MHT

**Figure 4:**
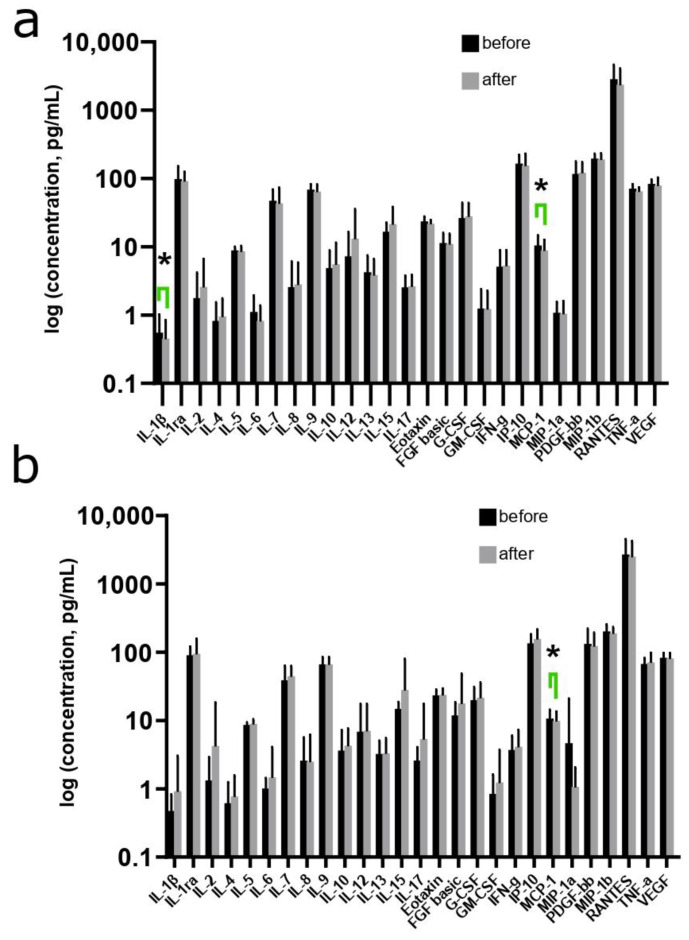
Changes in the level of cytokines in the blood plasma of women after 12 weeks of use of oral (**a**) MHT and transdermal (**b**) MHT. * *p* < 0.05 according to the Wilcoxon test.

The levels of 27 immune parameters in the blood plasma of women in both study groups were also measured using a multiplex blood test before and after 12 weeks of oral or transdermal MHT use (Figure 4a,b). According to the data presented, after 12 weeks of using both oral and transdermal MHT in women, there was a significant decrease in the level of MCP-1 from 10.23 (6.90; 13.96) pg/mL to 8.71 (5.39; 11.71) pg/mL (*p* = 0.022) and from 11.32 (9.53; 12.81) pg/mL to 8.72 (6.21; 12.29) pg/mL (*p* = 0.026), respectively. In addition, a significant reduction in IL-1β levels from 0.46 (0.26; 0.66) pg/mL to 0.26 (0.06; 0.56) pg/mL (*p* = 0.039) was observed after 12 weeks of oral MHT (Figure 4a).

## 4. Discussion

In this study, we compared for the first time the effect of two forms of MHT on the immune system by measuring a wide range of immunological blood parameters (subpopulation composition of lymphocytes, monocytes and NK cells) and the concentration of 27 cytokines and signalling molecules before and after 12 weeks of therapy. The 12-week period was not chosen at random but was justified by data from the literature [7,9,22].

Comparison of the results obtained with published data is only possible for certain aspects of the study. This is due to the limited number of similar studies in the literature. One of our key findings is that both oral and transdermal MHT have an effect on the subpopulation composition and expression of pro- and anti-inflammatory molecules on blood monocytes. Monocytes migrate from the bloodstream to the site of infection and inflammation where they differentiate into macrophages and dendritic cells [23]. Both dysfunction and, in some cases, dysregulation of an enhanced pro-inflammatory response have been demonstrated in studies of monocyte function in the context of immune aging. Some of them show that the proportion of so-called inflammatory monocytes, which express high levels of both CD14 and CD16 on the cell surface, increases in patients with age [23,24]. These data suggest that oral MHT has more pronounced effects on the immune status of patients. There is an apparent increase in the pro-inflammatory phenotype of various subpopulations of blood monocytes. In our work, we found a significant increase in both the percentage and intensity of staining of the total monocyte population positive for the pro-inflammatory marker CD80 when analyzing flow cytometry data from the blood of patients taking oral MHT. In the context of transdermal hormone therapy, our study found an increase in the percentage of CD206 cells among the monocyte subpopulations, while this indicator decreased with oral MHT, suggesting a possible protective role of transdermal MHT in relation to inflammatory diseases.

This study is the first to show that transdermal MHT results in a significant increase in T helper cells. CD4+ T cells are a large cell population that can be divided into several subtypes based on the spectrum of cytokines they secrete. In addition to classical T helper 1 and T helper 2, other subsets have been identified, including T helper 17, regulatory T cells, follicular helper T cells and T helper 9, which together fulfil multiple functions ranging from the activation of innate immune cells, B lymphocytes, cytotoxic T cells and non-immune cells to a critical role in suppressing the immune response [25]. The number of CD4+ T cells has been shown to decrease during menopause. Our results can be regarded as a positive effect of MHT, acting as a counterbalance to the decrease in the CD4/CD8+ cell ratio observed with increasing age compared to that of women in their reproductive period [26]. This phenomenon is probably related to the activation of memory T cells that colonize the reproductive tract via estrogen receptors [27,28]. To understand the mechanisms underlying this finding, it is necessary to study in more detail the changes in the composition of subpopulations of T, B and NK cells in the blood, as well as changes in local immunity.

Our study also showed a significant decrease in MCP-1 levels with different methods of MHT administration over 12 weeks. MCP-1 is one of the best-studied members of the CC chemokine subfamily. It is a 76 amino acid peptide that attracts and activates macrophages, controls vascular smooth muscle cells and can modulate cytokine production by T helper cells [29]. A number of studies have also shown a decrease in serum MCP-1 levels when MHT is taken with different delivery methods [30,31]. Yasui and colleagues noted a decrease in serum MCP-1 levels in postmenopausal women on the background of transdermal MHT for 12 months, but oral therapy did not show significant results [32]. Estrogens have also been shown to reduce the expression of MCP-1 mRNA in the injured arteries of ovariectomized rats [33].

A recent study noted a significant increase in MCP-1 levels in postmenopausal women (*p* = 0.022) compared to perimenopausal patients [34]. This is in line with current studies on the theory of immune aging and development of chronic low-grade inflammation in the elderly [35]. Tani et al. showed in their study that serum MCP-1 levels are higher in late menopause than in early menopause. MCP-1 levels showed a significant positive correlation with follicle-stimulating hormone (FSH) levels during the menopausal transition (r = 0.215, *p* < 0.01) [36].

MCP-1 is directly or indirectly involved in the pathogenesis of many diseases, in particular cancer, neuroinflammatory, cardiovascular, autoimmune (rheumatoid arthritis) and SARS-CoV-2 virus infection. MCP-1 is involved in the switch from rolling to adhesion of monocytes in the early stage of atherosclerosis [37]. Therefore, it is currently difficult to determine whether estrogen supplementation reduces the risk of cardiovascular disease in women with established atherosclerosis, and further clinical and laboratory studies are needed. Studies have shown that immune cells and cytokines are involved in all stages of atherosclerosis, from plaque formation to thrombosis, plaque rupture and heart attack [37]. In particular, MCP-1 is known to be a key modulator of atherogenesis. One of the main mechanisms by which estrogens suppress atherogenesis is thought to be the suppression of MCP-1 expression by vascular monocytes, leading to a decrease in macrophage recruitment to the arterial wall at the beginning of the plaque formation process [38]. Other mechanisms of estrogens are inhibition of MCP-1 expression by increasing in nitric oxide formation and suppression of endothelial adhesion molecules expression and vascular smooth muscle cell proliferation [39].

In addition, we observed a significant decrease in IL-1β level after 3 months of oral MHT, whereas transdermal MHT did not significantly change IL-1β levels. The introduction of exogenous estradiol (E2) reduced the expression of the pro-inflammatory cytokine IL-1β in peritoneal macrophages in both ovariectomized females and male mice [40]. In another study, it was shown that LPS stimulation resulted in reduced production of IL-1β and TNF-α in peritoneal macrophages of ovariectomized mice [41]. In Abrahamsen’s study, they showed a significant increase in the level of an IL-1 receptor antagonist (IL-1ra) in postmenopausal women during oral MHT. IL-1ra is known to specifically inhibit the action of IL-1β. Abrahamsen and colleagues did not find a statistically significant change in IL-1β levels, suggesting that levels of this cytokine were initially very low [42].

The fine mechanisms of the results obtained in current research are complex and have not yet been studied. It is possible to assume that the form of administration plays an important role. We link the mechanisms of MHT action on the immune parameters studied to the expression of estrogen receptors on blood cells. The complex relationship between sex steroid hormones and immunity suggests that MHT may have a pleiotropic effect on the immune system in menopausal women. Immune cells in the blood, including T lymphocytes and monocytes, express estrogen receptors [43]. Thus, when administered orally, estrogens are absorbed in the portal system and metabolized in the liver before entering the circulation. With the transdermal route of administration, estrogens enter the bloodstream directly through the vessels of the subcutaneous tissue and affect target organs before primary metabolism in the liver occurs. Transdermal delivery avoids first-pass metabolism in the gut and liver [44]. Advantages of transdermal delivery include the ability to deliver unmetabolized estradiol directly into the bloodstream, lower doses than oral drugs and minimal stimulation of hepatic protein production [45].

The effect of estrogens on innate immunity is based on the suppression of the production of proinflammatory cytokines IL-6, IL-1β and TNF-α by monocytes and macrophages, inhibition of chemokine production and prevention of immunocyte migration to the site of inflammation [17,46]. Estrogens also stimulate antibody production by B cells [47]. Estrogens modulate the expression of Th1 and Th2 cytokines, deactivate excessive inflammatory processes and restore homeostatic conditions, thereby potentially inhibiting the “cytokine storm” syndrome, and, as shown in COVID-19, reducing morbidity and mortality in women taking MHT [17,48].

Summarizing the effects of different MHT regimens on immune system parameters in menopausal women, we could conclude that oral and transdermal therapy generally have a beneficial effect, but transdermal MHT has a less pronounced effect on the immune system than oral MHT.

Among the limitations of our study, it should be noted that our results regarding the effect of different methods of MHT administration on immune system parameters should be considered as preliminary. Another of this study is that the study groups included both peri- and postmenopausal patients. In the future, it is worth conducting testing for each of the groups separately, expanding the sample size and testing different doses of the drugs.

## 5. Conclusions

We have shown that oral MHT leads to the activation and an increase in the percentage of monocyte subpopulations responsible for the inflammatory response and generally has a more pronounced effect on the immune parameters. At the same time, transdermal MHT led to an increase in the percentage of T helper cells as well as subpopulations of monocytes carrying the pro-inflammatory marker CD80. It can be cautiously assumed that the effect of the transdermal route of administration of estradiol in the composition of MHT on the parameters of the immune system may be preferential due to the ability to reduce the intensity of immunity compared to oral MHT. We have also shown that oral MHT leads to a decrease in the concentration of the pro-inflammatory cytokine IL-1β, which is likely to determine the protective effect of MHT in a number of inflammatory diseases. Further research is needed to understand the molecular mechanisms of the effect of different routes of MHT administration on immune system cells in order to individualize the therapeutic approach in women with different pathologies. Perhaps testing blood immune parameters or assessing immune status before prescribing MHT will become a routine step in clinical practice before choosing a patient management strategy.

## Figures and Tables

**Table 1:** Clinical and laboratory characteristics of patients before the start of oral and transdermal MHT.

Index	Oral MHT (*n* = 27)	Transdermal MHT (*n* = 23)	*p*
Age, years ^a^	51.4 (3.8)	50.2 (3.0)	0.356
BMI, kg/m^2 a^	23.9 (2.76)	24.0 (2.83)	0.766
Weight, kg ^a^	65.4 (7.61)	65.6 (7.13)	0.897
smoking ^b^	4/27 (14.8%)	3/23 (13.0%)	0.907
Duration of the period of absence of menstruation, months ^c^	13.0 (6.25; 27.0)	13.0 (6.0; 33.0)	0.811
The sum of points on the Greene’s scale ^c^	20.0 (14.0; 26.5)	19.5 (12.7; 27.2)	0.865
mild CS ^b^	6/27(22.2%)	6/23 (26.0%)	0.728
CS moderate ^b^	6/27 (22.2%)	6/23 (26.0%)	0.728
severe CS ^b^	15/27 (55.6%)	10/23 (43.5%)	0.547

Data are presented as ^a^ M(SD), where M is the mean value, SD is the standard deviation of the mean value (comparison was performed using Student’s *t*-test for independent samples); ^b^ absolute number and percentage in % (comparison performed using the Chi-square test); ^c^ Me (Q1; Q3), where Me is the median, Q1 and Q3 are the lower and upper quartiles (the comparison was made using the Mann–Whitney test). CS—climacteric syndrome.

**Table 2:** Dynamics of severity and frequency of occurrence of groups of menopausal symptoms in women after 12 weeks of oral/transdermal MHT use.

Index	Oral MHT (*n* = 27)			Transdermal MHT (*n* = 23)		
	Baseline	12 Weeks Later	*p*	Baseline	12 Weeks Later	*p*
Greene scale score *	20.0 (14.0; 26.5)	9.0 (4.5; 12.0)	0.001	19.5 (12.7; 27.2)	9.0 (5.0; 12.0)	0.001
Psycho-emotional symptoms (questions 1–11) *	11.0 (7.0; 14.0)	6.0 (2.0; 8.0)	0.002	9.5 (6.75; 14.0)	4.5 (2.0; 7.75)	0.012
Physical symptoms (questions 12–18) *	3.0 (0.5; 7.5)	1.0 (0.0; 3.00)	<0.001	3.0 (1.0; 6.25)	1.0 (0.0; 3.00)	0.022
Vasomotor symptoms (question 19–20) *	4.0 (2.5; 4.5)	0.0 (0.0; 1.0)	<0.001	4.0 (2.0; 6.0)	0.0 (0.0; 1.0)	0.000
Sexual symptoms (question 21) *	2.0 (1.0; 2.5)	1.0 (0.0; 2.0)	0.001	2.0 (0.0; 2.0)	1.0 (0.0; 1.0)	0.001

Data are presented as * Me (Q1; Q3), where Me is the median, Q1 and Q3 are the lower and upper quartiles (the comparison was made using the Wilcoxon test).

**Table 3:** Immune status of women using different forms (oral/transdermal) of MHT.

Index	Oral MHT (*n* = 27)			Transdermal MHT (*n* = 23)		
	Baseline	After 3 Months	*p*	Baseline	After 3 Months	*p*
Classical monocytes CD14++CD16−	85.4 (81.4; 91.5)	82.6 (74.0; 86.3)	0.020	81.3 (76.0; 84.8)	79.2 (75.6 82.8)	0.484
Non-classical Monocytes CD14−CD16++	7.29 (5.81; 9.88)	7.34 (5.84; 9.62)	0.5	8.61 (7.34; 9.60)	8.59 (7.38; 10.1)	0.131
Intermediate monocytes CD14+CD16++	7.55 (5.55; 13.60)	12.8 (8.30; 16.4)	0.023	5.76 (4.79; 6.63)	4.58 (2.79; 6.50)	0.094
T-helpers CD3+CD4+	64.8 (57.6; 69.5)	63.0 (56.5; 70.3)	0.387	65.5 (59.2; 70.9)	66.0 (59.9; 72.0)	0.005
Cytotoxic T lymphocytes CD3+CD8+	30.6 (25.9; 40.0)	33.7 (28.5; 40.7)	0.22	28.2 (25.4; 36.0)	29.3 (23.7; 37.6)	0.101
NK cells	5.76 (4.48; 14.5)	8.40 (3.60; 18.9)	0.045	4.83 (3.57; 7.77)	6.10 (2.85; 10.9)	0.58
B lymphocytes	6.90 (2.96; 15.4)	13.3 (3.56; 19.6)	0.045	4.95 (2.87; 7.67)	6.30 (2.00; 13.7)	0.941

Data are presented as Me (Q1; Q3), where Me is the median, Q1 and Q3 are the lower and upper quartiles (comparison was performed using the Wilcoxon test).

## Data Availability

The data presented in this study are available on request from the corresponding author due to patient privacy.

## Supplementary Materials

The following supporting information can be downloaded at: https://www.mdpi.com/article/10.3390/biomedicines12081892/s1, Figure S1. Gating strategy for flow cytometry. Gating strategy: on the graph of direct vs. side scatter, populations corresponding to lymphocytes and monocytes were isolated in terms of granularity and size. On the graph of expression of the pan-leukocyte marker CD45 against side scatter, populations corresponding to lymphocytes and monocytes were isolated. Then we made logistic gates for lymphocytes (A) and monocytes (B). Subpopulations of monocytes were determined by the expression levels of CD14 and CD16. Among lymphocytes, CD3 negative and positive populations were determined. T-killers and T-helpers by the distribution of CD4 and CD8 were detected among CD3 positive cells. Populations of B lymphocytes and NK cells were determined by the expression levels of CD19 and HLA-DR for the former and CD16 and CD56 for the latter among CD3 negative cells. Table S1. The Greene Climacteric Scale. Table S2. Antibodies and fluorophores compositions for flow cytometry analysis of the main blood cell subpopulations and pro-/anti- inflammatory (M1/M2) monocytes markers. Figure S2. Significant changes in the number of immune cells after 12 weeks of use (a) oral MHT (*n* = 27), (b) transdermal MHT (*n* = 22), * *p* < 0.05 according to the Wilcoxon test.
