# Supplementary material for: Effect of Menopausal Hormone Therapy on Cellular Immunity Parameters and Cytokine Profile
Source: Biomedicines. 2024 Aug 19;12(8):1892. doi: 10.3390/biomedicines12081892 (PMC11351925; doi:10.3390/biomedicines12081892)
Supplement: Supplementary file 1 [file biomedicines-12-01892-s001.zip › biomedicines-3128848-supplementary.pdf]

Supplementary material

Material and methods

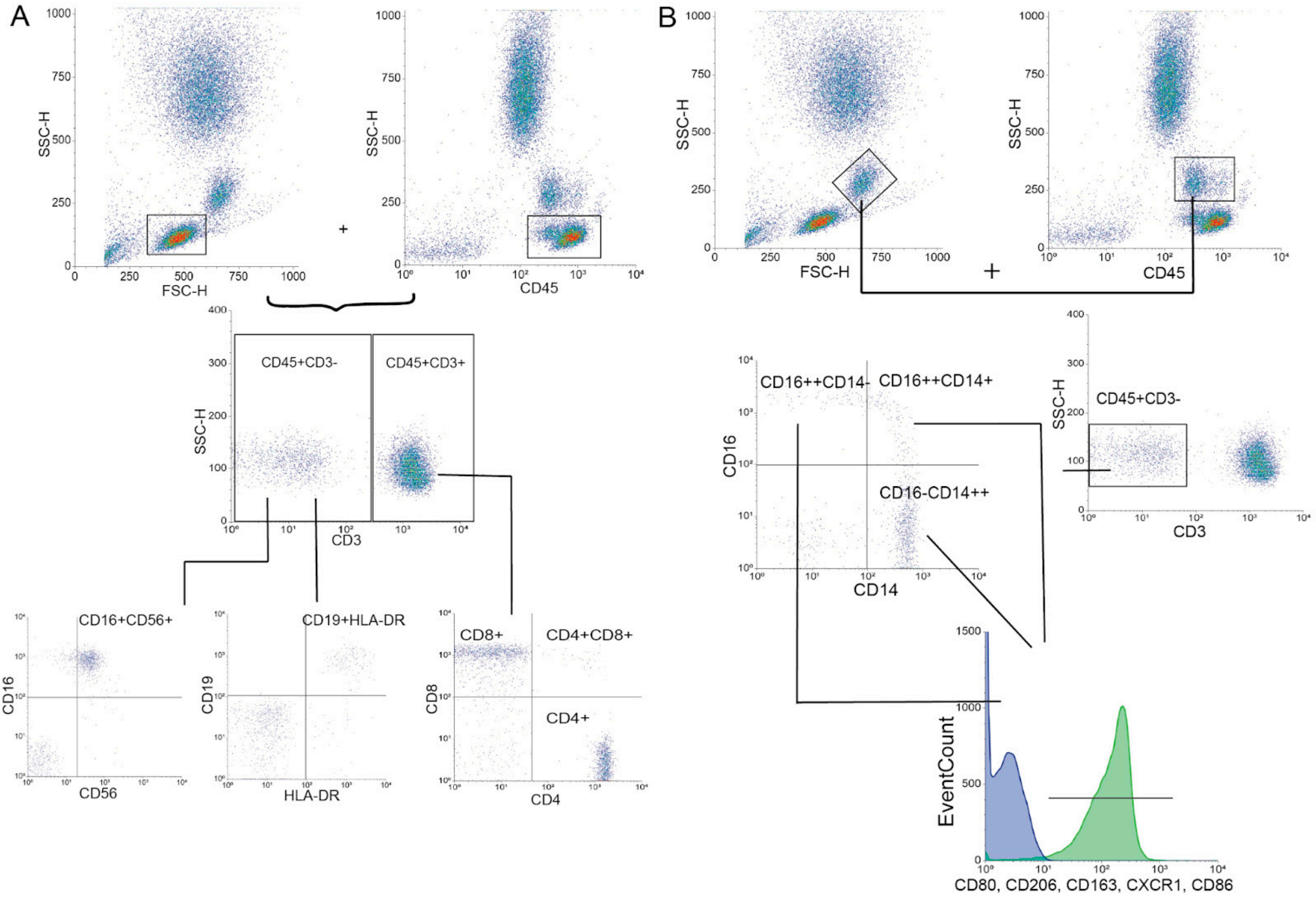

Figure S1. Gating strategy for flow cytometry. Gating strategy: on the graph of direct vs. side scatter, populations corresponding to lymphocytes and monocytes were isolated in terms of granularity and size. On the graph of expression of the pan-leukocyte marker CD45 against side scatter, populations corresponding to lymphocytes and monocytes were isolated. Then we made logistic gates for lymphocytes (**A**) and monocytes (**B**). Subpopulations of monocytes were determined by the expression levels of CD14 and CD16. Among lymphocytes, CD3 negative and positive populations were determined. T-killers and T-helpers by the distribution of CD4 and CD8 were detected among CD3 positive cells. Populations of B lymphocytes and NK cells were determined by the expression levels of CD19 and HLA-DR for the former and CD16 and CD56 for the latter among CD3 negative cells.

Table S1. The Greene Climacteric Scale.

Symptoms	Not at all	A little (1)	Quite often (2)	All the time (3)
Do you ever feel your heart beating quickly or pounding?				
Do you feel tense or nervous?				
Do you have difficulty sleeping? Either getting to sleep or waking up in the night?				
Do you often feel excitable?				
Are you anxious or experiencing panic attacks?				
Do you have difficulty concentrating?				
Are you tired or lacking in energy during the day?				
Have you experienced a lack of interest in things and activities?				
Do you often feel unhappy or depressed?				
Are you experiencing crying spells in your day to day life?				
Are you irritable with your family, friends and people you come into contact with?				
Dizziness, fainting				

Symptoms	Not at all	A little (1)	Quite often (2)	All the time (3)
Do you ever feel pressure or a tightness in your head?				
Do parts of your body feel numb?				
Are you experiencing headaches?				
Do you feel pains or aches in your muscles and joints?				
Do you ever experience a loss of feeling or numbness in your hands and feet?				
Do you ever have difficulty breathing?				
Are you experiencing hot flushes?				
Do you sweat in the night?				
Has your libido changed? Have you lost interest in sex?				

Table S2. Antibodies and fluorophores compositions for flow cytometry analysis of the main blood cell subpopulations and pro-/anti- inflammatory (M1/M2) monocytes markers.

Antibody	Fluorophore	Markers/Fluorophores Composition of Tubes	Cat Number
CD3	FITC	Killer and helper T-cells tube	130-113-138
CD4	APC-Vio770		130-113-789
CD8	PE		130-125-858
CD45	PerCPVio700		130-097-527
CD3	FITC	B-cells tube	130-113-138
CD45	PerCPVio700		130-097-527
CD19	APC		130-113-165
HLA-DR	PE		130-111-789
CD16	PE	NK-cells tube	A07766
CD56	FITC		130-114-549
CD3	APC		130-113-697
CD45	PerCPVio700		130-097-527
CD86	PerCP-Vio700	Classical, intermediate, non-classical monocytes tubes and pro- and anti-inflammatory markers	130-116-164
CD163	APC		130-097-630
CD14	FITC		130-110-518
CD16	PE		A07766
CD14	FITC	Classical, intermediate, non-classical monocytes tubes and pro-inflammatory marker	130-110-518
CD16	PE		A07766
CD45	PerCPVio700		130-097-527
CD80	APC		130-117-719

Antibody	Fluorophore	Markers/Fluorophores Composition of Tubes	Cat Number
CD14	FITC	Classical, intermediate, non-classical monocytes tubes and pro- and anti-inflammatory markers	130-110-518
CD206	PerCP-Vio700		130-104-129
CD16	PE		A07766
CX3CR1	APC		130-096-435
CD11b	APC	Classical, intermediate, non-classical monocytes tubes and pan - monocytes and pro-inflammatory markers	130-110-554
CD40	FITC		130-110-950
CD14	FITC		130-110-518
CD16	PE		A07766

Results

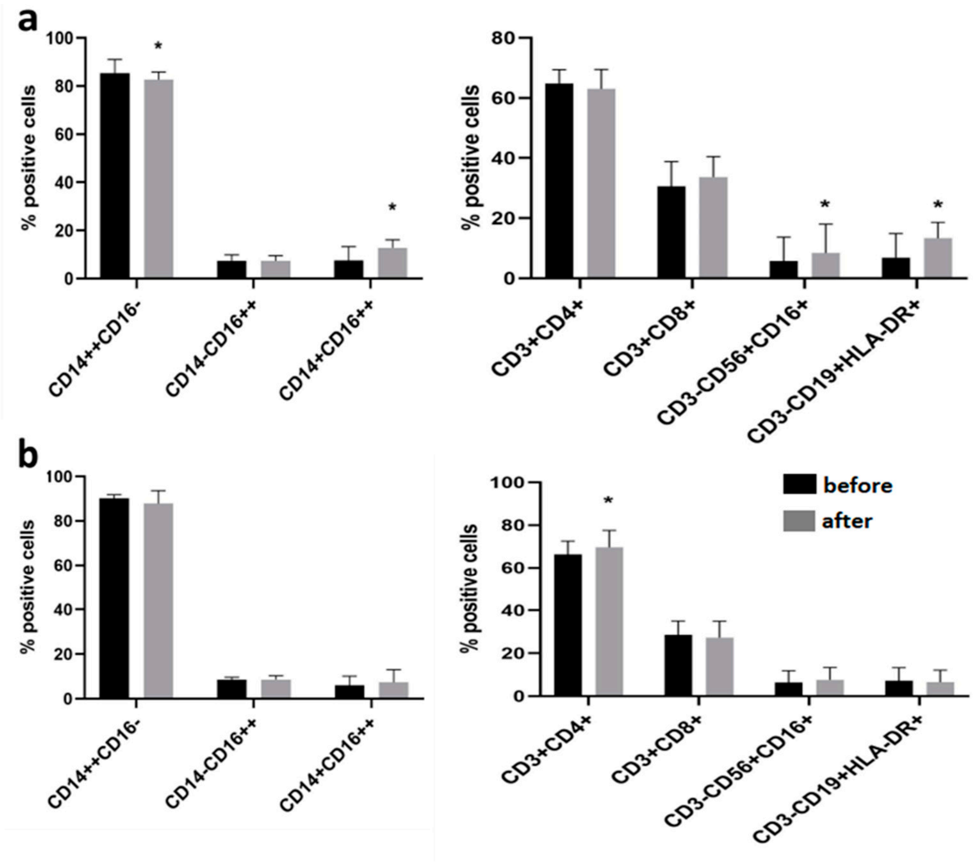

Figure S2. Significant changes in the number of immune cells after 12 weeks of use (**a**) oral MHT (n = 27), (**b**) transdermal MHT (n = 22), * $p < 0.05$ according to the Wilcoxon test.
